# Biometric Identification Based on Eye Movement Dynamic Features

**DOI:** 10.3390/s21186020

**Published:** 2021-09-08

**Authors:** Katarzyna Harezlak, Michal Blasiak, Pawel Kasprowski

**Affiliations:** Department of Applied Informatics, Silesian University of Technology, 44-100 Gliwice, Poland; michal.blasiak1@gmail.com (M.B.); pawel.kasprowski@polsl.pl (P.K.)

**Keywords:** eye movement, biometrics, nonlinear time series analysis, classification

## Abstract

The paper presents studies on biometric identification methods based on the eye movement signal. New signal features were investigated for this purpose. They included its representation in the frequency domain and the largest Lyapunov exponent, which characterizes the dynamics of the eye movement signal seen as a nonlinear time series. These features, along with the velocities and accelerations used in the previously conducted works, were determined for 100-ms eye movement segments. 24 participants took part in the experiment, composed of two sessions. The users’ task was to observe a point appearing on the screen in 29 locations. The eye movement recordings for each point were used to create a feature vector in two variants: one vector for one point and one vector including signal for three consecutive locations. Two approaches for defining the training and test sets were applied. In the first one, 75% of randomly selected vectors were used as the training set, under a condition of equal proportions for each participant in both sets and the disjointness of the training and test sets. Among four classifiers: *k*NN (*k* = 5), decision tree, naïve Bayes, and random forest, good classification performance was obtained for decision tree and random forest. The efficiency of the last method reached 100%. The outcomes were much worse in the second scenario when the training and testing sets when defined based on recordings from different sessions; the possible reasons are discussed in the paper.

## 1. Introduction

The security of computer systems, yet not only, is one of the most important issues of modern computer science. There are many solutions for protecting data as well as for user identification and authentication. They can be divided into three categories:

- something you know—e.g., passwords,
- something you have—such as tokens,
- something you are—biometric features.

Two-or multi-factor authentication, a combination of several authentication methods, has currently also become of interest.

In this paper, the attention is focused on biometric methods recognizing people based on human common, unique, permanent, and measurable physical or behavioural characteristics. The uniqueness of the trait means that it should be present in the case of all people. A given feature should be unique in the scale of the human population, and there will be no other who could use their features to impersonate another person. Permanence should guarantee that the feature remains unchanged throughout a person’s life, regardless of human aging or illnesses. The measurability of a feature should ensure that it will be measurable with the available technologies and estimation methods.

Among biometric traits used for identifying people, fingerprints, face shape, the eye’s retina, and iris can be mentioned. Utilizing physical ones has a disadvantage, that although challenging to forge, can be falsified with the usage of new technologies. It stems from the fact that they are relatively easy to access. For example, fingerprints can be taken from door handles or glasses and used by an unauthorized person to get into the protected system. Furthermore, even for voice characteristics, modern technologies can record and reproduce, with high accuracy, the voice of a person who has access to the resource. Such a recording can then be applied to break biometric security that utilizes a human voice for identification. Systems employing iris scanners can also be tricked with high-resolution iris photography [1].

These disadvantages entailed the search for behavioural features that are unique for each person, and at the same time, would be difficult to copy or repeat. Traits such as typing and walking meet these conditions as well as controlling mouse and eye movements. The last of the mentioned features were taken into consideration in this research.

The human eye movement is mostly not smooth and relies on quick and sudden jumps–saccades. Between jumps, fixations take place, focusing eyesight on an object, and during which the brain collects and processes information. Fixations last on average 200–300 ms, but their length can vary from tens of milliseconds to several seconds. Focusing on an object does not mean that the eye remains motionless. During the fixation, gentle eyeball movements can be observed, which are intended to keep the visible object in the centre of the retina, prevent the scene from fading or stabilize the eyesight while moving the head. These micromovements include microsaccades, drifts, and tremors. Microsaccades are the movement occurring at a typical rate of 1–2 Hz, with a small amplitude (less than $0.5^{\circ }$), high speed compared to drifts or tremors (15–50${}^{\circ }$/s), and a duration of 10–30 ms. Drifts are slow movements (with the amplitude less than $0.13^{\circ }$) with speed approximately equal to $0.5^{\circ }/$s, performed at a frequency below 40 Hz. The last of fixational movement types is micro-tremor with a small amplitude (approximately $0.00017^{\circ }$) and a frequency range of 40–100 Hz, reaching a top speed of $20^{\circ }$/s [2,3]. Because of these three movements’ existence, the eyes are never stable, and two additional quantities describe the fixation: the dispersion and speed of the eye.

Saccades are rapid eye movements aimed at setting the eye to the next fixation point. During saccades, no information is collected and processed. The saccade can be described by the following parameters: amplitude, speed, and duration. The saccades amplitude ranges from 4 to $20^{\circ }$, and the speed reaches 300–500${}^{\circ }$/s. Therefore, they are considered to be the fastest movements that the human body can make. The duration of saccades is most often 30–80 ms.

## 2. Related Work

Some studies devoted to the application of eye movements as biometric identification traits have been previously conducted. One of the first research in this field was described in the work [4]. This solution used the “jumping point” paradigm. 9 points in the form of a 3 × 3 matrix were displayed on the screen for 8 s. Nine participants were engaged in the experiment for whom 30 trials were recorded for the classification, giving 270 sessions in the data set. The first 15 cepstral coefficients were extracted from horizontal and vertical signals from both eyes. As a result, a 60-dimensional vector for each trial was obtained. The *k*-nearest neighbors (*k*NN) algorithm for *k* = 3 and *k* = 7, naive Bayes, decision tree, and support vector machine (SVM) were used as classifiers. The classification was performed using 10-fold stratified cross-validation. The best results were revealed for the *k*NN algorithm for *k* = 3. It achieved the average FAR (false acceptance rate) of 1.48% and FRR (false rejection rate) of 22.59%.

Further studies on biometric identification are described in the work [5]. Twelve people took part in the experiment, whose task was to observe static cross on the screen. Approximately 47 rows were received for each participant with a sampling rate 50 Hz. Such features as eye speed or differences in eye pupil diameter were calculated from the collected data. The combined features and each component separately were tested as a feature vector. For the classification, the *k*NN algorithm was chosen. The best results, which achieved the classification accuracy of 90% related to static feature–the distance between the eyes. For eye dynamics, the accuracy on the level 50%–60% were obtained for the difference in eye pupil diameter.

During the years 2012–2015, three eye movement verification and identification competitions were conducted. The first one [6] was held in 2012. Four data set types were employed: two uncalibrated and two calibrated. The stimulus was presented in the form of a jumping dot using the different layouts. Except for raw eye positional signal, only features related to the first and second derivate (i.e., velocity and acceleration, respectively) were considered, and *k*NN, SVM, and Bayesian network were used as classifiers. The experiment results showed that some unique noise present in the uncalibrated data sets might positively impact the classification accuracy. The other factor influencing results is time separating recording sessions. The smaller the time interval, the better the results. The second competition [7] organized in 2014 provided data collected during two sessions based on the faces observation. The results obtained by the participants revealed that there was no correlation between recognition rate and a sample’s length. The same regarded the familiarity of the face and the images themselves. However, this competition’s outcomes confirmed that the time interval between samples’ recordings had a significant impact on the classification accuracy. The third competition [8] taking place in 2015 provided four different data sets to allow verifying different parameters: different visual stimuli (random dot and text) and different time intervals between the recordings (three sessions–on average 30 min and 12 months apart). The recording device used for capturing the eye movements worked with a sampling rate equal to 1000 Hz. The competition participants mainly extracted statistical features from fixation and saccade profiles: position, direction, velocity, and acceleration. Among the methods used for classification, there were neural networks, SVM, and *k*NN. The competition results also showed that template aging had a more significant effect on recognition accuracy than visual stimulus type.

In [9] the authors conducted the eye movement-based biometric research in three sessions–30 min and one year apart, respectively. Eye movement data from the experiment were divided into fixations and saccades, and their statistical features were used for the classification purpose. Among them were the position, velocity, and acceleration profiles, as well as duration, dispersion, path length, and co-occurrence. The data set was registered with a 1000 Hz sampling rate and then decimated to 250 Hz. Two types of stimuli: random dot appearing on a screen and text, were utilized in the experiment involving 153 subjects for the first and the second session and 37 for the third one. In the classification process, two different Gaussian RBF networks were trained separately, and scores obtained from both networks were used to get the subject’s identity. The performance was approximately between 80% and 90%, dependent on the data set used.

There are also studies [10] in which the authors investigated the usage of saccades to verify a user from among other subjects. As stimulus jumping dot was used, for which 30 large horizontal amplitude saccades were obtained for 109 subjects. Eight features have been extracted from the signals: amplitude, accuracy, latency and maximum angular velocity, time to maximum velocity, maximum angular acceleration, and maximum deceleration. Four signals from every subject were gathered. One of them was taken for testing, and the other three for training. Subsequently, MLP and Radial Basis Function networks, Support Vector Machines, and Logistic Discriminant Analysis were utilized for the classification. Dependent on the method used, they obtained an accuracy of approximately between 63% and 85%.

The other research on this topic is described in [11]. The authors collected eye activity while reading the text. 40 participants took part in the experiment, divided into two groups of 20 people. Participants in the first group were presented with extracts from the Washington Post News articles, each with six texts, different for each person. The remaining 20 people read six excerpts from the papers that were the same for each participant. The data obtained from the eye-tracker was recorded with a sampling rate of 1000 Hz. The extracted features were divided into several types: fixation features (e.g., total duration and frequency of fixations), saccades ones (e.g., the average saccades duration, average saccade velocity, average horizontal amplitude of saccades), features of pupil reaction when reading the article, the frequency of changes in the pupil diameter. The classifying models were built utilizing such classifiers as Neural Network (multi-layer perceptron), random forest, and LMT (Logistic Model Tree). They were learned and tested using 10-fold cross-validation. The obtained accuracy, the combination of matching classes from different classifiers, was 95.31%, and the EER (Equal Error Rate) at the level of 2.03%. A comprehensive review of the related works in eye movement-based biometric identification can be found in [1].

The above-described studies yielded promising results, which, however, require further improvement in terms of identification efficiency. Additionally, in some research, the data sets were collected during a one-day experiment [10,11,12], which could facilitate subjects’ recognition. In others, the data were recorded with one-week or one-year intervals, yet unsatisfactory outcomes were obtained [6,7,8], or complex classification setup and stimuli were used [9,11]. It was the motivating factor for the exploration of new possibilities in this field.

The contribution of the research presented in this paper is introducing the novel approach for defining feature vectors. The proposed solution combines previously utilized quantities such as eye movement velocity and acceleration with characteristics evaluated using the nonlinear dynamic system analysis. To the best of the authors’ knowledge, such a solution has not been applied earlier in this field.

## 3. Materials and Methods

The currently presented studies were focused on developing a new approach for verifying users’ identity based on their eye movement signal. The task was realized in several steps. The first of them was to define feature vectors used for describing registered eye movement samples for each participant. Subsequently, some vectors obtained in such a way were applied to train a classifier to learn subjects’ eye movement behaviour. The remaining vectors part was utilized to verify the chosen methods efficiency meant as a proper recognition of samples as belonging or not to a user being identified. A detailed description of each research step was presented in the subsequent sections.

### 3.1. Data Set

**Figure 1:**
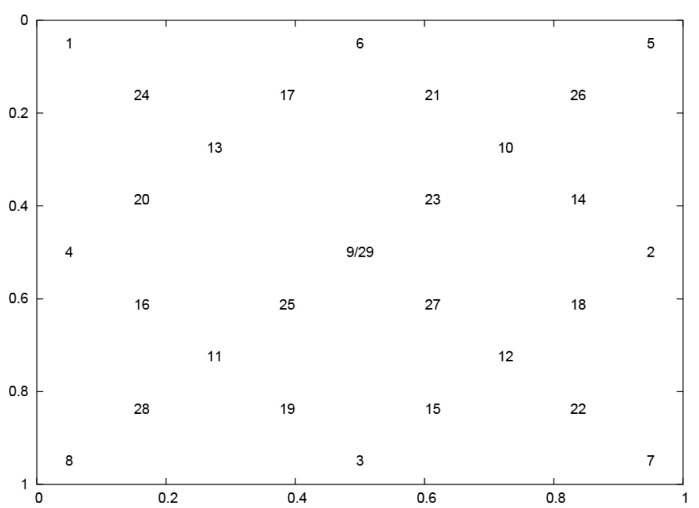
The layout of the jumping point positions.

The investigation was realized with the usage of data collected during an experiment involving 24 subjects. Their task was to follow with eyes the jumping point appearing in 29 positions on the screen (Figure 1). The 9th and 29th positions had the same coordinates. The stimulus layout was designed in such a way to ensure both covering a screen area evenly and to obtain varying lengths of saccadic movements. The stimulus was a dark circle of size $0.5\times 0.5^{\circ }$ pulsating–fluctuated in size–on the screen to attract participants’ attention. At a time, the point was visible only in one location and was displayed for 3 s. There were no breaks between the point’s subsequent presentations. After disappearance in one spot, it was shown at once in the subsequent one.

The experiment consisted of two sessions, during which the point was displayed in the same, predefined order. The sessions were held two months apart to avoid the learning effect that occurs when a participant is able to anticipate the next point position. Both sessions were conducted in the same environment: in the same room, on the same day of the week, and at the same time of the day. During both sessions, all participants had the same conditions. A room was equipped with artificial lighting, ensuring appropriate background for registering eye movements. Before each experiment, the participants were informed about the general purpose of the tests, after which they signed the consent form. As a result, 48 files were obtained, two for each participant.

The data was collected using the Ober Consulting Jazz-Novo eye-tracker, which records eye positions at a sampling rate of 1000 Hz [13]. The experiments used a 1280 × 1024 (370 mm × 295 mm) flat screen. The eye-screen distance was equal to 500 mm, and vertical and horizontal gaze angles were $40^{\circ }$ and $32^{\circ }$ respectively. The head was stabilized using a chin rest.

### 3.2. Feature Extraction

For the classification purpose, the first thousand registered samples after the stimulus presentation were considered for the analysis. This range includes three scopes of eye movement–the saccadic latency–the time needed by the brain to react to the stimulus position change, saccade and fixation. Subsequently, the chosen part of the eye movement signal was divided into 100-points segments; for each of them, the following 23 features were calculated:

- Maximum, minimum, average velocity, and the difference between maximum and minimum values (8 features)—for both eye movement directions,
- Maximum, minimum, average resultant velocity, and the difference between maximum and minimum values (4 features),
- Maximum, minimum, average resultant acceleration, and the difference between maximum and minimum values (4 features),
- Fourier transform (6 features)—Analysing eye movement dynamics, it was decided to change its representation from the time domain to the frequency one. The Fourier Transform was used for this purpose. Based on a signal x[n] for n=1,…,N, it creates a new sequence according to the following formula ([14]):
  (1) $$z\left[n\right]=\sum_{k=0}^{N-1}x\left[k\right]e^{\left(\frac{-i2\pi kn}{N}\right)}$$
  which is a frequency domain representation of a time-domain series–in this research the first derivative of horizontal eye position with respect to time. In the research, the Discrete Fourier Transform (DFT) implemented as the *fft* method in the {stats} package in R language was used. The output of this function is an array of complex numbers. The magnitude of each complex number represents the amplitude of a particular frequency for the overall data. The six first real values from the transform calculated for each segment were taken into account when defining the feature vector.
- The largest Lyapunov exponent (LLE) (1 feature)—The human eye can be treated as a nonlinear dynamical system, the characteristics of which can be studied with the use of methods for time series analysis [15]. One of such methods is the determination of the largest Lyapunov exponent (LLE), which allows assessing whether the nature of the system’s dynamics is regular or chaotic.

The estimation of the LLE is realized by the exploration of the system’s state space (Equation (2)), reconstructed based on the recorded dynamical system signal—a time series x(i),i=1,2,…,N.

(2) $$y\left(i\right)=\left[x\left(i\right),x\left(i+\tau \right),\cdots ,x\left(i+\left(m-1\right)\tau \right)\right],i=\left\{1,2,\cdots ,M\right\}$$

where M=N−(m−1)×τ. τ and *m* represent a time lag [16] and embedding dimension [17], two parameters crucial for a successful reconstruction of the system phase space.

In the presented studies as a time series the first derivative of horizontal eye position with respect to time was used. The time lag was calculated using the *Mutual Information* method and its implementation in the *mutual* function, included in the {tseriesChaos} package of R language. The second parameter–the embedding dimension–was determined by the *False Nearest Neighbours* method: *false.nearest* function from the {tseriesChaos} package. Then, the reconstruction of the system states was realized with the usage of the *buildTakens* function from the {nonlinearTseries} package.

**Figure 2:**
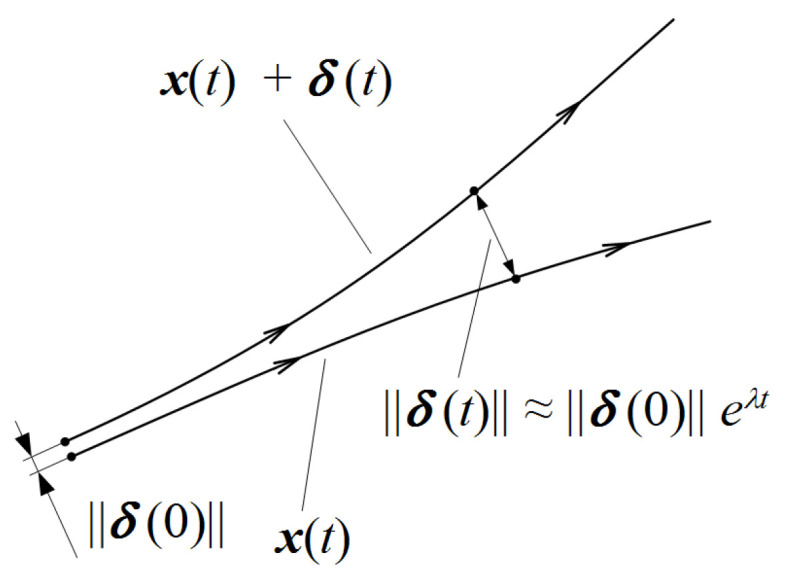
A path of a neighbouring pair of states in the phase space [18].

In the reconstructed phase space chaos can be observed by analysing the system’s behaviour during its evolvement. It is possible to follow the paths of neighbouring system states, which starting from close initial conditions, move to different states (Figure 2).

A pair [y(i),y(j)] of the nearest neighbours, starting close to one another in a chaotic system, diverges approximately at a rate given by the largest Lyapunov exponent λ [19]:

(3) $$\delta_{j}\left(i\right)\approx \delta_{j0}e^{\lambda (i\Delta t)}$$

where $\delta_{j}\left(i\right)$ is the Euclidean distance after *i* time steps, Δt is the sampling rate of the time series and $\delta_{j0}$ is the initial pair separation. Solving the Equation (3) by taking the logarithm of both sides, the largest Lyapunov exponent can be calculated as follows:

(4) $$\lambda \approx \frac{1}{i\Delta t}ln\left(\frac{\delta_{j}\left(i\right)}{\delta_{j0}}\right)$$

Equation (4) provides the way for evaluating λ for two specific neighbouring points over a specific interval of time. Thus, to approximate the Lyapunov Exponent for a whole dynamic system, it has to be averaged for all j=1,2,…,M, where M=N−(m−1)×τ. Negative Lyapunov Exponent values indicate convergence, while positive demonstrate divergence and chaos. The evaluation of the LLE was realized using *lyap_k* and *lyap* functions from the {tseriesChaos} package for each defined 100-points segment. A detailed description of the phase space reconstruction can be found in [20].

### 3.3. Classification Methods

The feature vectors described earlier were used in the classification process as an input to several classification algorithms. For the research purpose, the methods applied in the previously conducted studies [4,6,11,21] were utilized to check their performance on a different data set.

- *k*NN—a simple algorithm, assuming that similar objects exist in close proximity. It classifies an object in the feature space as belonging to a given class, taking into account the *k*-nearest neighbors of this object. The most commonly used distance between neighbours is the Euclidean one. In this research, *k* was set to 5 and 7.
- Decision Tree—a tree structure in which the internal nodes contain tests on attribute values (e.g., a feature is less or greater than a number). Depending on the result, another branch is selected, leading to the next node. The process is repeated until the leaf is reached. The class written in that leaf is assigned as the result of classification. From the most commonly used algorithms:
  - ID3 (Iterative Dichotomiser 3),
  - C4.5,
  - CART (Classification and Regression Trees).
  the last one, from the *scikit-learn* package in Python, was used in these studies [22].
- Random forest—algorithm using groups of decision trees for classification tasks. The final classification is made by the majority voting on individual decisions of trees included in the forest. The method implemented in the *scikit-learn* package, which calculates the final result as the average prediction probabilities of individual trees, was applied in these investigations [23].
- Naive Bayesian classifier–the method based on Bayes’ theorem.

(5) $$P\left(y|x_{1},...x_{n}\right)=\frac{P\left(y\right)P\left(x_{1},...,x_{n}|y\right)}{P(x_{1},...,x_{n})}$$

where *y* is a class and $x_{1},\ldots ,x_{n}$ is a feature vector. The Gaussian Naive Bayes classifier, assuming normal feature distribution, was used in the presented work.

### 3.4. Training and Test Sets

Two scenarios were explored during the presented research. In the first of them data recorded for one stimulus point were used to create one feature vector. 29 vectors were created for one session for each participant and 58 vectors for two sessions. In total, 1392 feature vectors were obtained with 10 segments; each contained 23 features. 75% of random vectors (1044) were used as training data, while the remaining 25% (348) were used as a test set. The vectors were split utilizing the *train_test_split* method from the *scikit-learn* package. The usage of the *stratify* parameter ensured avoiding a situation in which no vectors were found for given participants in the randomly selected training vectors, and all their vectors were included in the test set. The proportions of dividing the vectors into training and testing were the same for each participant, and the training and test sets were disjoint. The second scenario assumed that the feature vector was composed of the eye movement recordings for three consecutive points. The number of training vectors in this variant was equal to 972, while the number of test vectors equaled to 324.

## 4. Results

The assessment of the classification process was done in several steps and was confirmed with the usage of 10 randomly created training and test sets.

### 4.1. The Accuracy of the Vector-Based Classification

The first step of the investigation was to evaluate the chosen methods and defined features at the level of one vector. The five metrics were applied for this purpose. The first one was the accuracy, defined as the number of correctly classified vectors to the whole their number in the test set. Its averaged values for all four methods and both testes scenarios are presented in Table 1. It is visible that the worst results were obtained for naive Bayes and *k*NN (*k* = 5) in both variants; they ranged on average from 24% to 25% (28%) of the properly assigned vectors. Between *k*-values for *k*NN, the worse outcomes were obtained for *k* = 7; thus, only these for *k* = 5 will be discussed. The best classifier, in this comparison, was random forest, which achieved an accuracy of 72%; and for the second scenario, even 79%. The Decision Tree gave forty-seven percent of the correctly assigned vectors. Its performance slightly dropped to 46% when the extended feature vector was used.

In order to evaluate the classifiers performance and check in detail the predictions made for the individual classes, the Confusion Matrices were analyzed. They were evaluated based on True Positive (TP), True Negative (TN), False Positive (FP), and False Negative (FN) values. These quantities were used to determine the following metrics:

- Sensitivity, Recall, TPR–True Positive Rate:
  (6) $$\mathrm{sensitivity}=\frac{\mathrm{TP}}{\mathrm{TP}+\mathrm{FN}}$$
  Sensitivity informs to what extent the classifier is able to recognize instances correctly, taking into account conditions being tested.
- Specificity, TNR–True Negative Rate:
  (7) $$\mathrm{Specificity}=\frac{\mathrm{TN}}{\mathrm{TN}+\mathrm{FP}}$$
  Specificity measures the classifier’s ability to correctly generate a negative result for instances that don’t have the considered conditions.
- Precision, PPV–Positive Predictive Value:
  (8) $$\mathrm{Precision}=\frac{\mathrm{TP}}{\mathrm{TP}+\mathrm{FP}}$$
  Precision is the fraction of relevant instances among the retrieved instances
- F1 score–providing the balance between Precision and Recall
  (9) $$F1=2\times \frac{\mathrm{Recall}\times \mathrm{Precision}}{\mathrm{Recall}+\mathrm{Precision}}$$
  F1 score is defined as the harmonic mean of the precision and recall and thus takes both false positives and false negatives into account.

**Figure 3:**
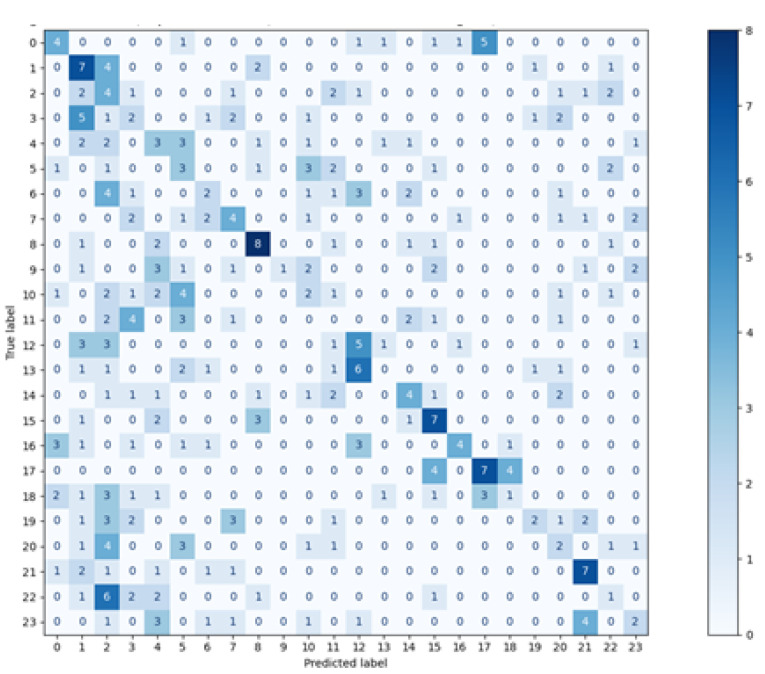
Confusion Matrix—*k*NN, *k* = 5.

**Figure 4:**
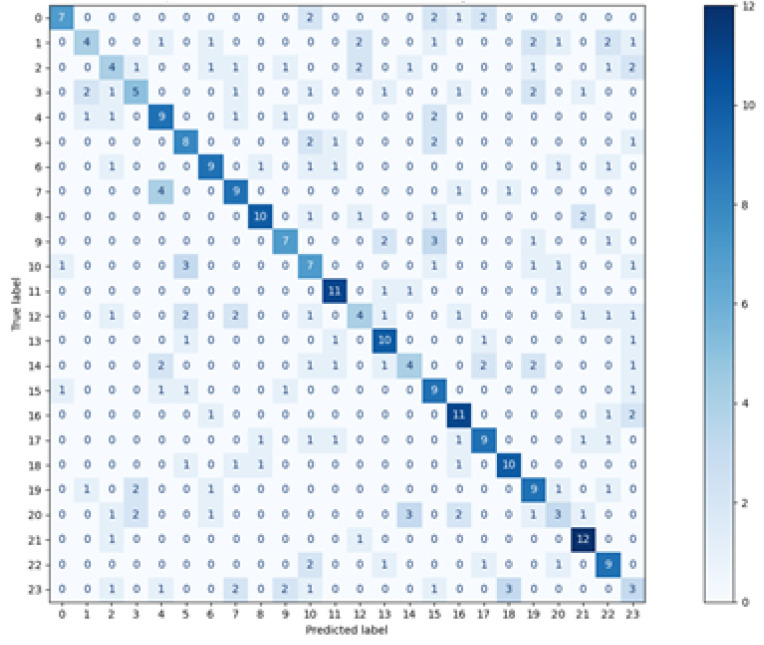
Confusion Matrix—Decison Tree.

**Figure 5:**
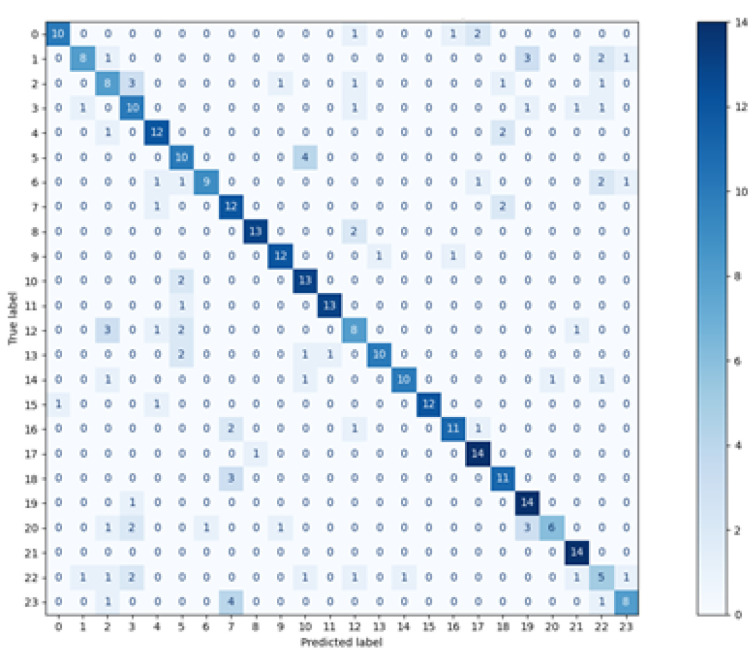
Confusion Matrix—Random Forest.

**Figure 6:**
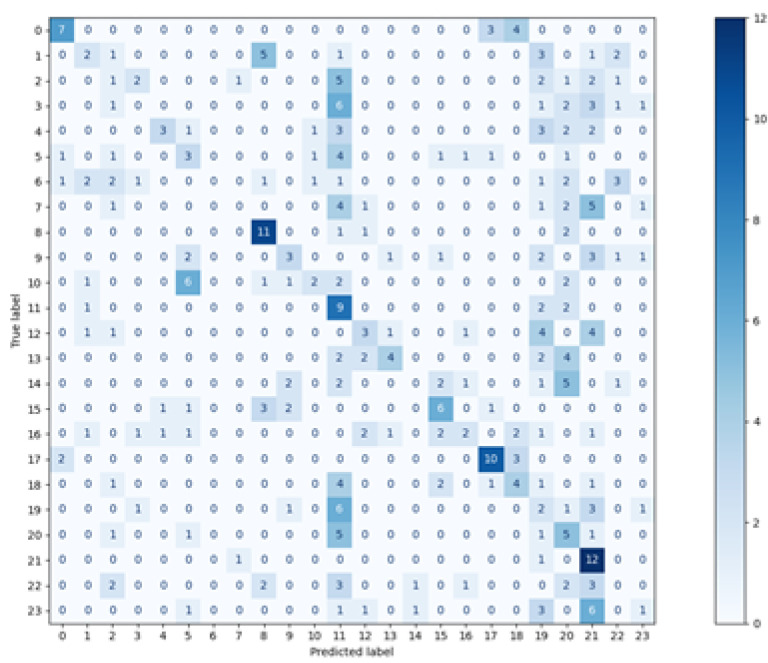
Confusion Matrix—Naive Bayes.

The matrices for all methods used are visible in Figure 3, Figure 4, Figure 5 and Figure 6. The real classes are presented on the Y axis, while the assignments made by the classifier are shown on the axis X.

When analysing the presented charts, it can be noticed that for most vectors, the classes assigned by two classifiers: Decision Tree and random forest, are located on the diagonal, which indicates a large number of true positive classifications (True Positive metric). There are small numbers of predictions beyond the diagonal. Both the matrices and the average metrics values, shown in Table 2, reveal that the random forest method featured the best performance. In contrast, the most considerable dispersion of data can be seen in the matrix relating to the *k*NN classifier. Similar matrices and metrics values were revealed for the second scenario, with three points used for defining one feature vector.

**Figure 7:**
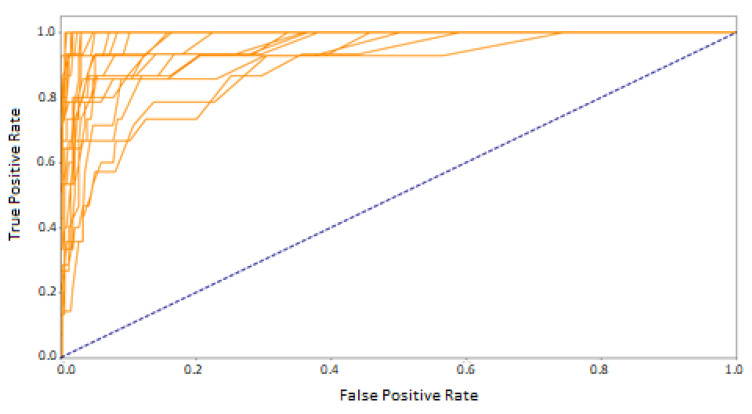
ROC for RF–24 classes. The train and test sets based on the mixed sessions.

Another step in the classification assessment was to study the Area Under Curve (AUC) calculated from the ROC curve. This value ranges from zero to one. The closer it is to 1, the classifier has a better TPR to FPR ratio. Because this curve summarizes the performance of a binary classification model on the positive class, it was decided to plot all 24 curves only for the best classifier, random forest (Figure 7). The figure shows that the curves are high above the dashed blue line, indicating good classification performance for all classes.

**Figure 8:**
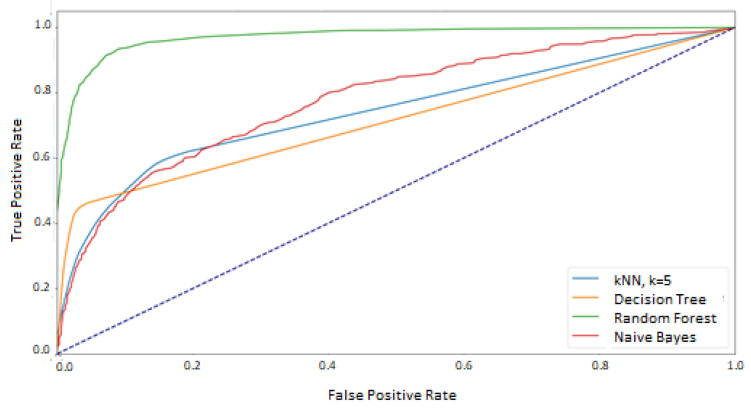
ROC–macro averages. The train and test sets based on the mixed sessions.

Additionally, since the test data was balanced, the macro averages for the classifiers were calculated as well as one ROC curve averaging results for each method was prepared (Figure 8). They show a significant difference between the random forest classifier and the other methods for which the area under the graph is similar.

The exact AUC values for macro means are presented in Table 3. They confirm that the *k*NN, decision tree, and naive Bayes classifiers yield similar performance. However, they are less effective than the random forest classifier.

### 4.2. Majority Voting for the Final Classification Result

The final classification result was to provide a class for each subject taking part in the experiment. It was realised by determining the dominant from classes assigned to the participant’s vectors from the test set. The obtained results are presented in Table 4. The percentage of correctly classified subjects is much higher than the classification accuracy presented in Table 1 for one feature vector. The weakest classifier in this comparison is Naive Bayes, which correctly assigned classes for only 9 out of 24 subjects when the scenario with one point per vector is considered. In the second analyzed variant, the worst results produced the *k*NN method with 10 subjects properly identified. The Decision Tree yielded an outcome with almost twice the percentage of subjects than the classification accuracy at the level of the single vector. The random forest once again turned out to be the best algorithm from the studied group; its effectiveness for the first scenario on average was 100%, which means that it correctly classified all subjects. However, for one out of ten classification runs, it recognized properly 23 out of 24 subjects. In the second scenario, such a situation took place twice.

### 4.3. Results for the Classification Based on Separated Sessions

**Figure 9:**
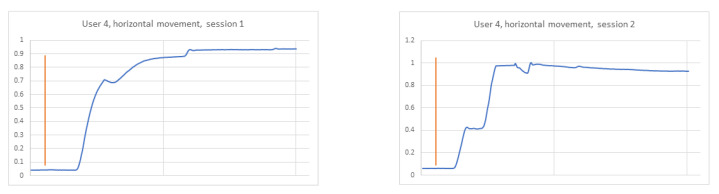
Horizontal signal–user 4 session 1 on the **left**, session 2 on the **right**.

**Figure 10:**
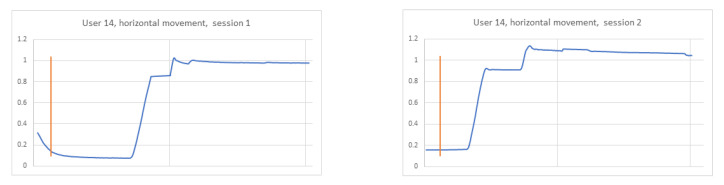
Horizontal signal–user 14 session 1 on the **left**, session 2 on the **right**.

**Figure 11:**
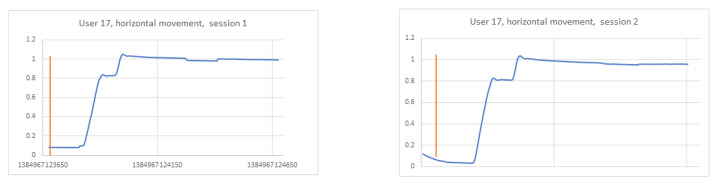
Horizontal signal–user 17 session 1 on the **left**, session 2 on the **right**.

In the previously conducted analysis, the training set was created based on recordings collected during two sessions of the experiment to provide more data for the learning phase. The achieved results were encouraging; thus, the next step of the studies was performed, aimed at verifying a strategy in which the train and test sets were created based on each session independently. When analysing signals for three different participants in both sessions, it became clear that this task would be more challenging. It was checked whether the data differed between participants, and at the same time, whether they were similar to each other for the same participant in two different sessions (Figure 9, Figure 10 and Figure 11). The same stimulus position was selected for the comparison (namely, the second). In the figures, the orange line marks the timestamp at which the stimulus was displayed.

In the figure relating to participant 4 (Figure 9), it can be seen that the signals for the selected point are different for each session. The charts for participant 14 (Figure 10) are similar to each other in both sessions, which may contribute to the proper identification of this subject. However, the 14th and 17th (Figure 11) participants’ graphs are similar, which in turn may lead to incorrect classification of these users.

**Figure 12:**
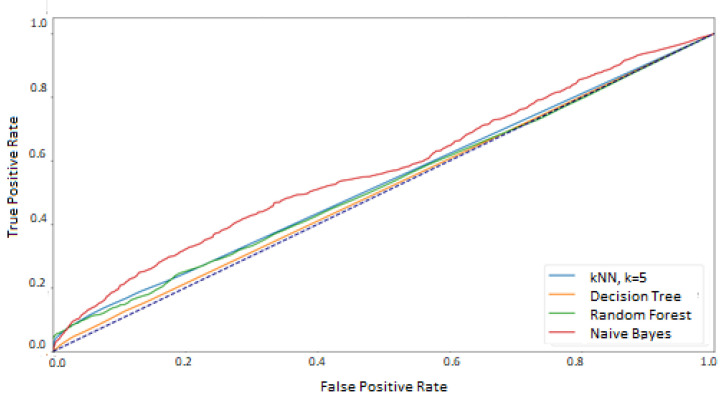
ROC–macro averages. The train and test sets based on the different sessions.

**Figure 13:**
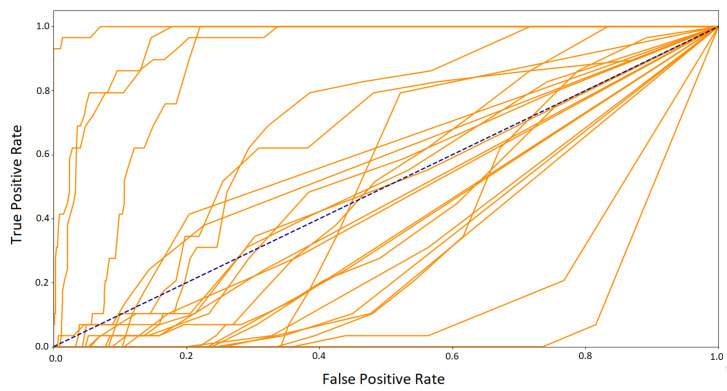
ROC–RF for 24 classes. The train and test sets based on the different sessions.

The results confirmed the expectation regarding the difficulty of the classification. The macro averages for the classifiers and ROC curves averaging outcomes (Figure 12) revealed that the classification quality is low, close to the random choice (blue dashed line). The ROC curves for the random forest classifier for all classes presented in Figure 13 show a large discrepancy between the classes. It can be noticed that for some of them, the outcomes are close to the ideal classifier, and for others, worse than the random classification.

The final classification results based on the majority voting from the classes assigned to the participants’ feature vectors are shown in Table 5.

Summarizing the results obtained for the configuration in which one vector was created based on one point, and the first session was used as training data, and the second session as test data, it can be noticed that the quality of the classification for each method is low. The average classification performance was close to the random classifier. The Naive Bayes classifier was the most efficient among the four methods, whereas the Decision Tree was the least.

## 5. Discussion

The results presented in the previous chapter were obtained as a result of the implementation of two approaches for defining the training and test sets. In the first approach—where the data used in the training phase came from randomly selected feature vectors from both experimental sessions—the high and good classification efficiency was achieved for two methods: random forest and Decision Tree. However, the random forest was undoubtedly the best classifier in this comparison, which was proved on several levels:

- at the macro-mean level for the results averaged over all classes (Table 3 and Figure 8)
- at the class level (Table 4 and Figure 7)
- at the level of a single feature vector (Table 1 and Table 2 and Figure 4 and Figure 5)

The reason for such good results may be the fact that the classifiers, as the training set, obtained data from two experimental sessions, which gave a more diverse description of the participants’ eye movement characteristics. The subsequent argument for better results, than in the second variant, is the higher number of training vectors, constituting 75% of all recorded signals. Both factors could make the classification of the test set elements easier.

In the second approach, when the data obtained in one session was included in the training set and the test set contained signals from another one–the number of training vectors and the representation of the eye movement characteristics of specific people decreased. The graph of ROC curves for the random forest (Figure 13) shows that the classifier, in such a configuration, obtained good results for some participants, which is the encouraging finding. Unfortunately, there was a group of classes with much worse outcomes. It suggests the existence of differences in those subjects’ eye movement signals registered in the first and second sessions. They may result from several factors.

One of them is that most participants during the first session took part in an experiment involving eye-tracking technology for the first time. It could introduce a certain unnaturalness to the eye movement, which differed from the typical characteristics of a given person. During the second session, participants were more experienced, which might reflect their natural eye behavior, different from the previous one. The other factor could be the experimental environment which, despite making much effort to be identical in both sessions, could introduce some unnoticeable differences in eye movement registration. Finally, the participants’ disposition on a given day could also slightly change the dynamics of their eye movements. These two last factors will be present in many eye movement experiments. Therefore, mixing data from various sessions in training sets could result in a better fit of the model to the changing eye movement characteristics in different conditions. However, to not over-fit a classifier, it has to be ensured that the same data are not repeated in training and test sets. Such rules were applied in the first part of these studies’ experiment. The best solution would be to carry out several sessions and provide the classifier with data containing various eye movement characteristics in such amount that will ensure high performance of the classification on a test set, the content of which was collected in an independent session. Such research is planned as future work.

The comparison of the results obtained in this research with those presented in the previous works confirmed the good efficiency achieved for the random forest classifier for the approach, in which the mixed sessions were used for training and testing. In [5] the authors obtained the accuracy of 60% for the delta pupil size when observing a cross stimulus displayed in the middle of the screen. The data was collected within one session for four cross observations with sampling rate 50 Hz. This set during classification was split, taking signals registered for three observations as training vectors and one for the test set for all 12 engaged participants. The *k*NN method with *k* = 3 was used.

From the work presented in [12], for the comparison purpose, the results for data gathered with 1000 Hz sampling rate, for 32 participants, were analyzed. In these studies, the experiment consisted of four 1-min recordings regarding a textual stimulus, was conducted. From collected signals, the fixation and saccade profiles were used to define feature vectors. Such a data set was divided for training and testing, ensuring the disjointness of both sets. During the classification, the accuracy of approximately 83% was achieved for the random forest method.

Slightly better results were obtained in [24] where the authors proposed a multi-stimulus and multi-algorithmic approach. The data set was constructed from over 3-min recordings of the observations of four stimulus types by 320 participants. Two sessions 20 min apart were conducted, data from which was used to create the disjoint training and test sets. The fusion of the outcomes for all stimuli and three classifying methods resulted in an accuracy equal to 88.6%.

A similar accuracy, between 80% and 90% was obtained by the authors in [10]. They utilized the “jumping point” paradigm in the experiment consisting of separate measurements recorded in succession with a sampling rate of 250 Hz, each lasting 60 s. Three of them were used as the training set and one for testing. The saccade characteristics were applied as feature vectors for 109 subjects.

The subsequent studies to be compared were described in [11]. The presented experiment consisted of two parts, during which 40 participants read two different texts. From the signals recorded with a sampling rate of 1000 Hz, fixation, saccade, pupillary response, and spatial reading features were extracted. The Logistic Model Tree and Random Forest methods were trained and tested using a 10-fold cross-validation. The combination of outcomes from both classifiers yielded an accuracy of 97%.

The second part of the comparison regarding the results coming from the classification based on disjointed sessions with other research applying this approach [6,7,8,9] confirmed that it is more challenging for most studies. However, the outcomes obtained in this research need significant improvement. As mentioned, the first step planned for this purpose is to collect more sessions for each subject with a meaningful time interval and apply methods for noise removal. Furthermore, a detailed exploration of the newly introduced features will be performed. Finally, introducing other measures evaluated by nonlinear time series analysis is also considered.

## 6. Conclusions

The studies presented in this paper focused on biometric identification with the usage of eye movement signal. New features, based on nonlinear time series analysis, were introduced for defining vectors used in the classification. The investigations revealed that they served well when data collected during two experiment sessions were used for creating both the train and test sets. Good performance was achieved, especially by the Random Forest method–better than in the other similar studies yet obtained in shorter experiment with simpler stimulus and larger intervals between sessions.

However, weak accuracy was obtained, where the first session was used for training and the second for testing. Thus, further research is planned in this area. At first, more sessions will be conducted, and other features representing nonlinear eye movement dynamics will be considered for the classification purpose.

## Figures and Tables

**Table 1:** The averaged accuracy obtained on the feature vector level for the four classification methods. The standard deviation is provided in brackets.

Method	Accuracy	Accuracy
	One Point per Vector	Three Points per Vector
*k*NN, *k* = 5	24% (1%)	24% (2%)
Decision Tree	47% (3%)	46% (3%)
Random Forest	72% (3%)	79% (2%)
Naive Bayes	25% (3%)	28% (2%)

**Table 2:** The averaged metrics obtained on the feature vector level for the four classification methods. The standard deviation is provided in brackets.

Method	Sensitivity	Specificity	Precision	F1
*k*NN, *k* = 5	0.24 (0.016)	0.97 (0.0007)	0.26 (0.015)	0.25 (0.021)
Decision tree	0.47 (0.034)	0.98 (0.0015)	0.47 (0.032)	0.46 (0.033)
Random Forest	0.71 (0.023)	0.99 (0.0010)	0.73 (0.025)	0.71 (0.025)
Naive Bayes	0.25 (0.025)	0.97 (0.0010)	undefined *	undefined *

* means that TP + FP, was equal to zero.

**Table 3:** The Area Under Curve averaging results for each method, calculated for two scenarios and averaged over ten classification runs. Values in brackets represent standard deviation.

Method	AUC-One Point per Vector	AUC-Three Points per Vector
*k*NN, *k* = 5	0.73 (0.013)	0.75 (0.010)
Decision Tree	0.72 (0.018)	0.72 (0.014)
Random Forest	0.97 (0.003)	0.98 (0.002)
Naive Bayes	0.78 (0.014)	0.78 (0.009)

**Table 4:** The averaged accuracy obtained on the subject level for the four classification methods. The train and test sets defined based on vectors mixed from two sessions. Values of standard deviations are presented in brackets.

Percentage: Number of Correctly Classified Subjects		
Method	One Point per Vector	Three Points per Vector
*k*NN, *k* = 5	50% (7%):12 (2)	41% (8%):10 (2)
Decision tree	92% (4%):22 (1)	83% (6%):21 (2)
Random Forest	100% (1%):24 (0)	99% (2%):24 (0)
Naive Bayes	38% (4%): 9 (1)	49% (7%):12 (2)

**Table 5:** The accuracy obtained on the class level for the four classification methods– the train and test sets built from the different sessions.

Method	Percentage (Number) of Correctly Classified Subjects
*k*NN, *k* = 5	12% (3)
Decision Tree	4% (1)
Random Forest	12% (3)
Naive Bayes	17% (4)

## Data Availability

Data set used in the research will be available online at http://kharezlak.pl/eyetracking.html, from the 1st of October 2021.
